# “We just needed to open the door”: a case study of the quest to end solitary confinement in North Dakota

**DOI:** 10.1186/s40352-021-00155-5

**Published:** 2021-10-18

**Authors:** David H. Cloud, Dallas Augustine, Cyrus Ahalt, Craig Haney, Lisa Peterson, Colby Braun, Brie Williams

**Affiliations:** 1 Amend, University of California, San Francisco, School of Medicine, 490 Illinois Street, Floor 8, UCSF Box 1265, San Francisco, CA 94143 USA; 2 Department of Psychology, University of California, Santa Cruz, 1156 High Street, Santa Cruz, CA 95064 USA; 3 North Dakota Department of Corrections and Rehabilitation, 3100 Railroad Avenue, P.O. Box 1898, Bismarck, ND 58502-1898 USA

**Keywords:** Solitary confinement, Prison reform, Correctional health

## Abstract

**Abstract:**

Solitary confinement is a widespread practice in US correctional facilities. Long-standing concerns about the physical and mental health effects of solitary confinement have led to litigation, legislation, and community activism resulting in many prison systems introducing policies or implementing legal mandates to reduce or eliminate its use. Yet little is known about the nature and effectiveness of policies that states have adopted to reduce their use of solitary confinement and exactly how various reforms have actually impacted the lives of people living and working in the prisons where these reforms have taken place.

**Methods:**

We conducted an embedded case study, analyzing changes in policies and procedures, administrative data, and focus groups and interviews with incarcerated persons and staff, to describe the circumstances that led to changes in solitary confinement policies and practices in the North Dakota Department of Corrections and Rehabilitation (ND DOCR) and the perceived impact of these changes on incarcerated persons and prison staff. .

**Results:**

North Dakota’s correctional officials and staff members attributed the impetus to change their solitary confinement policies to their participation in a program that directly exposed them to the Norwegian Correctional Service’s philosophy, policies, and practices in 2015. The ensuing policy changes made by North Dakota officials were swift and resulted in a 74.28% reduction in the use of solitary confinement between 2016 and 2020. Additionally, placements in any form of restrictive housing decreased markedly for incarcerated persons with serious mental illness. In the two prisons that had solitary confinement units, rule infractions involving violence decreased at one prison overall and it decreased within the units at both prisons that were previously used for solitary confinement. Although fights and assaults between incarcerated people increased in one of the prison’s general population units, during the initial months of reforms, these events continued to decline compared to years before reform. Moreover, incarcerated people and staff attributed the rise to a concomitant worsening of conditions in the general population due to overcrowding, idleness, and double bunking. Both incarcerated persons and staff members reported improvements in their health and well-being, enhanced interactions with one another, and less exposure to violence following the reforms.

**Conclusions:**

Immersing correctional leaders in the Norwegian Correctional Service’ public health and human rights principles motivated and guided the ND DOCR to pursue policy changes to decrease the use of solitary confinement in their prisons. Ensuing reductions in solitary confinement were experienced as beneficial to the health and wellness of incarcerated persons and staff alike. This case-study describes these policy changes and the perspectives of staff and incarcerated persons about the reforms that were undertaken. Findings have implications for stakeholders seeking to reduce their use of solitary confinement and limit its harmful consequences and underscore the need for research to describe and assess the impact of solitary confinement reforms.

## Introduction

Solitary confinement is the practice of confining incarcerated persons in a small cell for approximately 22 h per day. This generic term can be applied to different types of prison housing assignments, such as restrictive housing, administrative segregation, disciplinary segregation, and protective custody (Haney, 2018a). In addition to the deprivation of meaningful social contact, persons who are incarcerated in these units often have limited or no access to programming and restrictions on the amount and nature of their visits and personal property (Haney, 2020). Generally, they are only permitted to leave their cells for showers or to recreate alone in a small closed-in or caged area. People with serious mental illness, cognitive impairment, those who are LGBTQ, and members of racial and/or ethnic minority groups are overrepresented in solitary confinement (Bertsch et al., 2020; Reiter & Blair, 2015; Schlanger, 2012; Ryan & DeVylder, 2020). The stated reasons for placing someone in solitary confinement range from punishment and involuntary or voluntary “protective custody,” to safeguarding the “safety and security” of the institution (which may include isolating persons with verified or perceived gang affiliation). The amount of time spent in solitary confinement may extend from days to decades (Bertsch et al., 2020) and varies depending on a range of factors, including agency policy, the type of solitary confinement unit (e.g., disciplinary versus administrative), among others. The most recent survey of state correctional systems reported that between 55,000 to 62,500 people in U.S. state prisons were in solitary confinement on any given day in 2019 (Bertsch et al., 2020), another study found that 20% (320,000) are exposed to administrative or solitary confinement at least once annually (Beck, 2015).

The use of solitary confinement is considered by leading professional medical societies (e.g., the American Medical Association, 2016, the American Public Health Association, 2013, the American Psychiatric Association, 2017, the National Commission on Correctional Healthcare, 2016, and the World Medical Association, 2019) and international human rights organizations (e.g. the United Nations, Human Rights Watch) to be a pressing public health and human rights concern (Ahalt & Williams, 2016; Cloud et al., 2015; Gottschalk, 2015; Méndez, 2019). Although some authors have questioned the extent to which solitary confinement has an adverse impact on health and well-being (e.g., Morgan et al., 2016; Gendreau & Labrecque, 2018), a number of studies have found that solitary confinement can produce or exacerbate negative mental health symptoms (Grassian, 2006; Haney, 2018b; Rhodes, 2004; Smith, 2006), is associated with increased self-harming and suicidal behavior (Kaba et al., 2014; Lanes, 2009, 2009), increased morbidity (including PTSD), physical harms (Morgan, 2017; Strong et al., 2020; Williams & Ahalt, 2019), and even mortality following prison release for persons who have spent time in solitary confinement (Brinkley-Rubinstein et al., 2019; Hagan et al., 2018; Strong et al., 2020; Wildeman & Andersen, 2020). These finding are in line with a large body of research outside of correctional contexts which demonstrates that social isolation, social exclusion, and loneliness have profoundly debilitating effects on physiological and psychological functioning (e.g., Haney, 2020; Leigh-Hunt et al., 2017; Williams & Ahalt, 2019).

The concerns about the health-related impact of solitary confinement (based in part on these studies and also on testimonials of people who have been subject to it), have led to decades of litigation, legislation, and community activism (Fettig, 2019, Schlanger, 2020) to reduce its use. Increasing numbers of state prison systems are introducing policies or implementing legal mandates intended to reduce or eliminate the use of solitary confinement (Bertsch, L., et al. 2020). Yet little is known about the types of policies that states are adopting to reduce or eliminate solitary confinement, whether these policies succeed in doing so, or how these policy changes are experienced by people who live or work in the affected prisons.

As a first step in understanding these issues, we describe and evaluate one multi-pronged approach designed to reduce use of solitary confinement undertaken by the North Dakota Department of Corrections and Rehabilitation (“ND DOCR”). These reforms were inspired by and based largely on the principles and practices of the Norwegian Correctional Service. We detail the nature of the specific policy changes made by the department, assess the degree to which they were implemented, and analyze some of their reported impacts on the health and well-being of incarcerated persons and staff.

## Methods

### Study design and setting

We employed an embedded case study design to describe and assess the nature and effects of changes in the ND DOCR’s use of solitary confinement, including trends in violence (lower-level “fights” and higher-level “assaults” as defined by correctional officer write-ups in the administrative disciplinary records) before and after the policy changes. We chose the embedded case study approach because it is well-suited for in-depth assessment of the development, implementation, and impact of interventions and policy changes in real world settings through researcher-practitioner partnerships (Scholz & Tietje, 2002; Petersilia, 2008).

The men’s solitary confinement housing units in the ND DOCR are located in two prisons—the maximum-security North Dakota State Penitentiary (NDSP), and the medium security James River Correctional Center (JRCC). NDSP is a 1500-person facility built in 1883. JRCC is a much smaller and newer prison; built in 1998, it holds approximately 365 incarcerated people. North Dakota’s women’s prison is operated by a private entity and not part of this case study. Our analysis of the ND DOCR’s reform efforts focused on all of the housing units that prison officials targeted for solitary confinement reform in the two men’s facilities (NDSP and JRCC), including disciplinary and administrative segregation units, restrictive housing, and a housing unit designated for people with significant mental health conditions experiencing acute mental and behavioral health crises. Our goal was to assess the ways that the department’s policy changes affected the units *they* defined as solitary confinement; the possibility that different forms of isolated housing may produce different effects on incarcerated people (e.g., Mears et al., 2019, 2019) was beyond the scope of this study.

### Data and data analysis

Our analysis draws on semi-structured interviews with correctional staff, focus groups with incarcerated persons, and the department’s administrative data. Participants (both staff and incarcerated people) were asked to describe the nature and effects of the policy changes that were implemented in the period after North Dakota correctional officials participated in a correctional reform program that immersed them in the principles, policies, and practices of the Norwegian Correctional Service in 2015 (Ahalt et al., 2020; Amend, 2021). In addition, it includes a descriptive analysis of the department’s administrative data, including disciplinary, diagnostic, and housing records that we used to assess changes in the use of solitary confinement units and rates of disciplinary infractions involving violence (defined as fights or assaults) before and after the reforms began within these two prisons.

#### Semi-structured interviews with prison staff and incarcerated persons

Our semi-structured interviews with ND DOCR correctional staff and focus groups with incarcerated persons were conducted in February 2020. To describe the perceived impacts of policy changes on staff, we conducted 14 semi-structured interviews with a purposive sample of correctional leaders, clinicians, case managers, and line staff who were directly involved in creating and/or implementing the reforms. Interviews were conducted in person or via a videoconferencing call.

We then conducted five focus groups with a total of 32 incarcerated persons (19 from NDSP and 13 from JRCC) to learn about their experiences with solitary confinement before and after the policy changes, and the impact of those changes on their health and well-being. Focus group eligibility included having been incarcerated in North Dakota for at least 3 years and having experienced solitary confinement. Researchers sent a recruitment flyer and interview script to case managers who compiled a list of interested participants. Potential participants were randomly selected on the day the focus groups were held. Focus groups were designed to have an average of 6 participants; three groups were held at NDSP (the larger facility), and two groups were held at JRCC (the smaller facility). Participants were provided an overview of the case study and written copies of the informed consent in advance. Researchers read the consent form aloud in person and obtained verbal consent; one person elected not to participate. Participants were not provided monetary incentives. Focus groups were held in a private conference room inside the prisons, ranged in length from 90 to 120 min and were digitally recorded. Staff interviews and focus groups with incarcerated persons were transcribed and uploaded to NVivo for qualitative coding and thematic analysis.

#### Departmental policies and administrative data

We conducted a descriptive analysis of departmental policies and administrative data beginning in January 2010, based on guidance from ND DOCR officials who identified this as a point in time when the department began planning to expand its use of solitary confinement. We defined the “post-reforms” period as beginning in January 2016 when department leaders returned from their first visit to Norway, implemented immediate releases from administrative segregation, began revising their solitary confinement review protocols and disciplinary rules, and first established a housing unit for people who engage in violence as an alternative to solitary confinement. We ended our analysis of the post-reform observation period on December 31, 2019, prior to the COVID-19 pandemic.

We analyzed ND DOCR’s operating policies over time (e.g., rules governing use of disciplinary housing units, resident handbooks, officer job descriptions, action plans for solitary confinement reforms, training materials, data analyses, and presentations describing the reforms) to document changes in the department’s use of solitary confinement, inform interview and focus group guides, and contextualize our analysis of administrative data.

We used the department’s administrative records of disciplinary actions filed by correctional officers to quantify and determine trends in solitary confinement sanctions. We also analyzed housing records (“movement files”) to describe admissions to, and to compare lengths of stay in, different housing units over time.

Using the ND DOCR’s administrative data we then described the average rates and counts of correctional officer reports of violent infractions before and after the reforms began. For this analysis, we included all disciplinary events recorded in the department’s administrative data in which officers charged a person with a rule violation involving some degree of violent behavior. All “violent infractions” include any correctional staff-issued rule violation charges for behaviors involving physical violence (regardless of severity). The administrative data further characterize interpersonal violence into two levels of severity, these are: (1) “Fights Among Residents” which are considered less severe acts and include charges for “fighting” defined as “punching, kicking, striking or wrestling with another person in an aggressive manner” and (2) “Assaults between Residents” which are considered more severe acts and include “an attack upon any other person … causing mental or bodily injury, or causing offensive contact.” We also reported acts categorized as an “Assault and battery on staff” defined as any “attack upon a volunteer, employee, officer, or official of the ND DOCR [including] sexual assault, causing mental or bodily injury, or causing offensive contact.”

Next, to further assess the relationship between changes in the use of solitary confinement and behavioral infractions for interpersonal violence, we calculated Incidence Rate Ratios (IRR) between the monthly rates of solitary confinement and each indicator of violence using negative binomial regression with fixed effects. We chose this technique because our outcome(s)—monthly rates of each indicator of violence—were not normally distributed across the observation period and were overdispersed (Hilbe, 2011). We included a fixed effect in the model to account for unobserved heterogeneity between the units in the two different prisons (NDSP and JRCC), since these institutions are distinct in many ways that we could not measure (Alison, 2002).

Additionally, we conducted an interrupted-time series (ITS) analysis and Prais-Winstein Regression to assess whether there were significant changes in trends of fights and assaults before and after ND DOCR began implementing three key components of their reforms: enactment of new disciplinary policy policies to limit placements in solitary confinement for most rule infractions; enhancements to the staffing and clinical services for people with mental health needs assigned to the Special Assistance Unit (SAU) at JRCC; and establishment of the Behavioral Intervention Unit (BIU) as a last resort housing area primarily for people who commit serious assaults. Each of these reforms is described in more detail in later sections. We selected May of 2016 as the point of intervention for ITS based on consultation with ND DOCR leadership confirming the start of each aforementioned reform. ITS is widely-used for evaluating the effects of laws, policies, and interventions on health outcome, because it allows for examining differences in slope and intercept between the series data before and after a policy change or intervention occurs, while including autocorrelation terms (Biglan et al., 2000; Bernal et al., 2017).

We acknowledge that retrospective administrative data is an imperfect way to study violence in prisons. For example, charging officers may have different thresholds for classifying an incident as more severe (in this case causing “mental injury *or* bodily injury”) versus less severe. We therefore reported differences in the rates of each of these levels of charges both collectively and separately over time when relevant. All quantitative analysis was conducted using STATA Version 16. This study was approved by the Institutional Review Board at the University of California, San Francisco.

## Results

### Solitary confinement in North Dakota 2010–2015

Like many prison systems in the United States, North Dakota’s prison population increased dramatically over the last several decades. Between 1980 to 2010, the number of people incarcerated in the ND DOC increased sevenfold and the state’s prisons were plagued by overcrowding and escalating violence (Bertsch et al., 2020). In 2012, a prison expansion nearly doubled the number of long-term solitary confinement cells at NDSP, and the median length of stay in solitary confinement increased from 109 days in 2012 to 136.5 days by the end of 2013. According to a former warden, after the expansion:

It was like that old adage. If you build it, they will come. It was almost night and day. Anybody who caused any type of trouble that disrupted the norm for general population… they were put into segregation [solitary confinement].

Both incarcerated peoples and staff said that the expansion led to a sharp punitive turn in ND DOCR’s culture. One staff noted there was “a big shift into more command, control, and lock down,” and that violence and unrest increased. An incarcerated person recalled, “there was crazy fights and chaos all the time...There used to be stuff happening every single day here. There was group tension, actual hatred.”

Before 2016, correctional officers in North Dakota had discretion to impose solitary confinement for a wide range of behaviors. Incarcerated people described its use for “petty” activities (disobeying an order, tattooing, talking back to an officer, having unauthorized property). One recalled: “if you even tried to have a debate with an officer that didn’t like you, then they could just take you to the hole.” A staff member acknowledged his own tendency to use solitary confinement in scenarios where there was a minor disruption. He stated “if somebody was a nuisance person, or whatever the case may be, I would push to have them put back in segregation just because I didn’t want to deal with them. I had so many other people I had to deal with.”

Lengths of stay in solitary confinement were indeterminate. Staff were required to review each person’s eligibility for return to the general population every month for the first 90 days and bimonthly thereafter. Staff and incarcerated people  relayed that decisions regarding release from solitary confinement were subjective without clear requirements. One incarcerated person remarked, “in the old system, you’re back there until they are done being mad at you. The door ain’t ever open. There wasn’t a concrete way to work your way out.” Another stated, “They used to just leave you back there for months, just see you when they saw you … get to you when they got to you... It was rough.”

Many staff and incarcerated persons  who were interviewed about this period in ND DOCR’s history characterized the solitary confinement units at both of its main prisons as dehumanizing, volatile, and traumatizing. Officers acknowledged that they were not trained to build positive relationships with incarcerated people nor expected to do so. One ND DOCR leader said that officer trainings reinforced the notion that getting to know an incarcerated person actually jeopardized staff safety:

We just continued to go down that path which ended up being like ‘don’t talk to inmates. That’ll be the number one way for you to make sure that you’re safe. That became the training mantra. Don’t trust them. It became us versus them from the very onset.

An NDSP officer lamented “I felt the best I could do was [act] like a flight attendant … meeting their basic needs, giving them food, toilet paper, or whatever they needed, but not helping them.” In addition, officers were taught that their safety depended on keeping people in solitary confinement or fearful about being placed there. Officers and incarcerated persons alike described their interactions as infrequent, hostile, and aggressive. An incarcerated person who was in solitary confinement during this time recalled:

They treated guys like shit. It was just constant. They’d ignore them … . Just walk by them. They used to yell, lie to your face, “Oh, I’ll talk to [treatment staff]. They’re going to come and see you.” And they don’t come back. It sends us into a damn trauma state.

Persons housed in these units were locked in their cells at least 23 h per day, and access to clinical care, educational opportunities, and rehabilitative programming was limited or non-existent. One man who spent several years in solitary confinement during this period at NDSP recalled, “There were times that I sat in the hole for such a long period of time … It was fucking psychological torture. I can’t think of anything worse.” Another man, in solitary confinement in his early twenties, still grapples with the emotional toll: “When you’re a kid, you’re free and innocent and the whole world’s ahead of you. Then all of a sudden, you’re in a hole for months and months. It scarred me and changed my life forever.”

During this time, JRCC’s Special Assistance Unit (“SAU”), a housing area established for people with serious mental health conditions, devolved into what one clinical administrator described as “solitary confinement by another name.” Staff acknowledged that SAU patients received sporadic and inconsistent mental health services, and that clinicians spent more time reacting to crises than providing therapeutic services. People who decompensated in solitary confinement at NDSP were frequently moved to the SAU at JRCC for more clinical assistance. However, “the expectations were pretty unclear … and a lot of times if people acted out, we had a punitive prison response, versus a therapeutic response.” Staff described the environment as stressful and traumatic for them as well. A clinical psychologist recalled:

For years and years and years, there was just a ton of trauma in that unit. We had many people who tried to kill themselves, a lot of self-harm, a lot of staff injuries. Some very, very major and serious assaults on staff that left people very disabled. And just a lot of consistent crises.

One SAU clinician noted that there was:

“Lots of infighting, lots of blaming other people for things going wrong, lots of distrust between staff. There were lots of amygdalar [reactive] decision making, avoidance, and all the other things that happen when you’re just saturated in chronic toxic stress.”

Another psychologist remembered working conditions in the SAU as “just eating up people alive” and causing burnout and turnover among clinicians and security staff.

### Early solitary confinement reform efforts (2012–2014)

In 2012, ND DOCR leadership enrolled several staff members in National Institute of Corrections trainings to explore approaches to solitary confinement reform. This led to the department adopting individualized behavioral health plans and a “level-system” intended to help transition people out of administrative segregation at NDSP and the SAU at JRCC. However, these National Institute of Corrections-inspired changes did not reduce the use of solitary confinement. Instead, solitary confinement punishments actually increased from 3.30 sanctions per month per 100 incarcerated personss in 2012 to 4.72 in 2014, and the median length of stay in NDSP’s administrative segregation increased from 109 days in 2012 to 136.5 days in 2013.

### Involvement with amend/Norway and a new approach to solitary confinement reform

In August 2015, officials from the ND DOCR began participation in a novel cross-cultural exchange program led by Amend at the University of California San Francisco (UCSF) in collaboration with the Norwegian Correctional Service. Amend is a public health focused program that aims to reduce the debilitating health effects of US prisons on incarcerated people and staff (Ahalt et al., 2020; Amend, 2021). Initially begun as a joint program of UCSF faculty and the Prison Law Office in California, it later formalized as a partnership between UCSF faculty and the Norwegian Correctional Service. Amend provides U.S. prison systems with educational curricula, immersive training programs, and technical assistance for correctional officials and staff members to initiate and implement changes in policies and practices that are based on the Norwegian Correctional Service principles of dynamic security (fostering positive interpersonal relations between staff and incarcerated persons), normalization (creating correctional conditions that resemble as closely as possible the community conditions to which an incarcerated person will return), and progression (continuously moving incarcerated persons to less restrictive environments) (Table 1).

**Table 1:** Key Principles of the Norwegian Correctional System (Labutta, 2016)

**Dynamic Security**	Positive interpersonal relationships between correctional staff and incarcerated people are essential for safety and wellbeing in prison; the investment of time, resources, and services that nurture human relationships is essential to reducing the risk of conflict, disruption, and violence.
**Normalization**	The goal of prison is to return a “better neighbor” to society, therefore living conditions inside a correctional institution should resemble life outside the facility to the maximum extent possible; incarcerated people retain all other human rights aside from the loss of liberty. Policy, practice, and the architecture of a prison should promote individual autonomy and responsibility for making choices necessary in a community setting (e.g., shopping for groceries, preparing meals, earning income and managing finances, enrolling in school or vocational training, and participating in civic duties and activities)
**Progression**	During incarceration people should gradually advance toward greater freedoms in their
	living circumstances, responsibilities, and environments as they progress from admission to reentry into society.

Norway’s correctional system is often heralded for its humanistic philosophy, beginning with the assumption that the deprivation of liberty—going to prison—is the punishment imposed by a court for having committed a crime, not the occasion for imposing more punishment. In addition, the Norwegian Correctional Service also assumes that the function of a prison is to promote rehabilitation, health, and successful community return through intensive rehabilitative services and a well-trained, professional correctional workforce (Høidal, 2019; Justice, N. M. o.,, and Police, T, 2018). Despite criticism for its use of solitary confinement during pre-trial detention—a controversial practice among a number of Scandinavian countries—the Norwegian correctional service employs solitary confinement far less frequently, and for a much shorter duration, than is the norm across U.S. prisons (Høidal, 2019; Norwegian Parlimentary Ombudsman, 2019).

ND DOCR officials cited their immersion in the public health-focused values, principles, and practices of the Norwegian Correctional Service as a catalyst that inspired their efforts to end solitary confinement. A former warden recalled, “before Norway, we were talking about changing restrictive housing and administrative segregation [solitary confinement]. There were already things happening, but this was just that bomb that landed in the middle of all of it.” Another ND DOCR official explained that lessons learned in Norway inspired immediate actions upon their return:

There was a sense of urgency that we couldn’t just hang on and talk about it. You hate to be part of a system that does harm to people we are supposed to be helping. We just needed to open the door, put people back into the [general] population because we were using [solitary confinement] for purposes that we said we weren’t … punishment, not safety.”

**Fig. 1:**
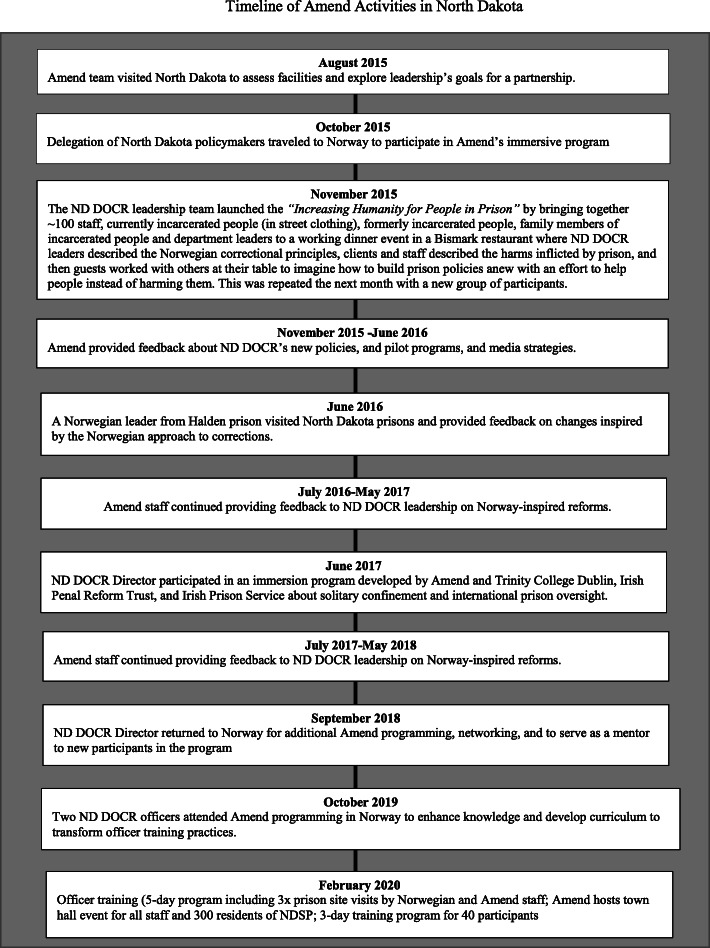
Timeline of Amend Activities in North Dakota

**Fig. 2:**
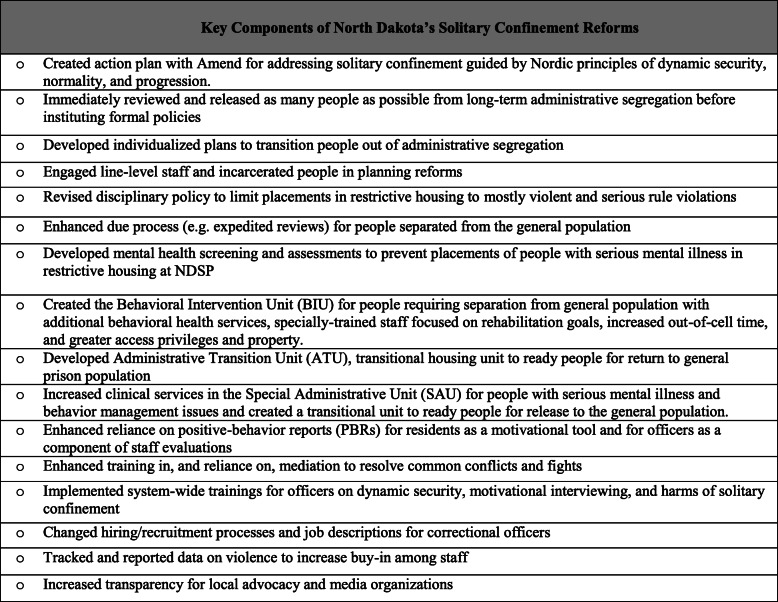
Key Components of North Dakota’s Solitary Confinement Reforms

ND DOCR named their Norway-inspired initiative to change correctional culture *Increasing Humanity for People in Prison.* (See Fig. 1 for timeline of Amend-led activities and Fig. 2 for a list of policy changes included in ND DOCR’s initiative.) One prison official described learning about the health consequences of solitary confinement as a motivating factor for initiating reforms,

Now I think that longer term staff acknowledge that some residents have been permanently damaged by being locked up in restrictive housing for so many years, after listening to the personal stories of some of our residents talk about what it had done to them. It made me cry, thinking what we had done to people before we made these changes.

### Solitary confinement reforms in North Dakota (2015–2019)

ND DOCR officials took a number of specific steps designed to implement significant changes in their correctional culture and to reduce their reliance on solitary confinement. We discuss each of them below, including what staff and incarcerated people reported to us about the impact that changes in these policies and practices had on their health and well-being.

#### Immediate releases from solitary confinement

In the weeks immediately following their 2015 immersion experience in Norway, ND DOCR officials returned 30 people from long-term solitary confinement to the general population. A warden recalled, “we literally just opened up the door. We met with the guys and told them we don’t have a plan, but our success is based on your success, and we need to figure out something.” Over the ensuing weeks, clinical staff spent more time building rapport with incarcerated people, learning about their histories, needs and goals, and developing plans for them out of solitary confinement. A clinical administrator recalled:

At the beginning, it was basically just running trial by error … All we wanted to do was for staff to be able to work with the people in segregation, more one-on-one, more frequently, and offering more life skills and more how to handle conflicts and manage emotions. That’s where we started.

Not surprisingly, perhaps, a number of staff members were initially uncomfortable with these changes. A security officer described their concerns about the rapid release of people from long-term solitary confinement: “I thought the roof was going to fall off. Things were going to blow up and be so dangerous, but that really wasn’t the case.” An incarcerated personremembered “I was literally in administrative segregation when it transitioned from the old way. They started turning it into more of a one-on-one and started to pull us out of our rooms. It was just the beginning. That was a tipping point.”

#### Creation of a transition unit

In October 2015, top ND DOCR officials recruited staff members who had experience helping incarcerated people prepare for community reentry to develop a unit that prepared persons to transition from solitary confinement to the general population. One officer recalled that behavioral health and security staff prioritized building rapport with people on the unit, gaining an understanding of each person’s concerns and challenges, and developing individualized plans for moving them back to a dormitory setting. Next, ND DOCR accelerated the review process to once a week. Staff reported that more frequent reviews, and increased releases, had a noticeably positive effect on interactions between staff and incarcerated people. A case manager remarked that these changes provided “a little bit more light at the end of the tunnel.. . That’s when I definitely felt a big change.”

#### Changes in disciplinary policy

In 2016, ND DOCR officials codified these changed practices into actual policies designed to limit the use of solitary confinement, increase reliance on mediation to resolve disputes, and to rescind rules that had little correctional purpose but could nonetheless result in disciplinary sanctions (e.g., requiring people to tuck in their shirts). Disciplinary infractions that can result in solitary confinement in the ND DOC are now limited primarily to serious acts of violence resulting in injury. Officials also changed the nature of the units in which persons could be isolated from the general population. For example, at JRCC, persons who commit violent rule violations or manifest an acute behavioral health need are sent to one of two program- and treatment-intensive specialized units—a Behavioral Intervention Unit (“BIU”) and a Special Assistance Unit (“SAU”) (both of which are described in further detail below).

In an effort to modify the atmosphere inside these treatment-oriented units, ND DOC officials greatly expanded an already existing initiative in which staff were encouraged to file positive behavior reports (“PBRs”) that recognized incarcerated people for displaying empathy and kindness to others and/or prioritizing their own educational or clinical goals. One clinician described PBRs as a

systematic way of recognizing people and the good things that they do and the good parts of themselves, to help them invest in themselves more. Staff now have to sit down and talk about the good things and that actually shifts staff culture a lot … the way that people see their jobs here … and breaks through some of that negative mindset that can happen when you’re constantly dealing with situations where you feel a lack of the total control and turn to fear-based responding.

In fact, as one measure of the shift in focus from punishing negative behaviors to acknowledging positive ones, NDSP officers issued more than twice as many PBRs as rule violations in the BIU (493 PBRs versus 225 rule violations) between 2015 and 2019.

#### Improved mental health screening and Services for People with serious rules violations

The ND DOCR established a “checks and balances” process to limit admissions to BIU (the Behavioral Intervention Unit at NDSP) and the SAU (the Special Assistance Unit at JRCC) that included mental health screens and required officials to review placements within 2 workdays. Correctional officials must now decide between three possible outcomes within 48 h: immediate release from restrictive housing, referral to behavioral health staff for assessment for placement in the BIU program, or transfer to the SAU.

In addition, to facilitate the enhanced emphasis on treatment, oversight of the SAU (the Special Assistance Unit at JRCC) at JRCC was changed from custody staff to a licensed clinical psychologist. Policy now dictates that clinical and security staff collaborate to stabilize incarcerated persons'’ mental health conditions and expedite their return to general population. Two “human relations counselors” provide therapeutic services on the unit (e.g., coaching, group counseling, crisis de-escalation, and observation reassessments), and incarcerated people are offered individualized clinical care, group counseling, and congregate activities (e.g., art therapy, television, games) at least daily.

Staffing credentials and staff training within the SAU at JRCC were changed as well. Officers are now assigned to unit based on their temperament and commitment to working with people with acute mental health needs and receive training to assist with delivering the individualized behavioral health plans developed by JRCC clinicians. Annual SAU staff training was redesigned to focus on identifying and responding to self-harm, in addition, supervisors and clinicians now receive specialized crisis assessment training, and the SAU’s crisis intervention team is now comprised of both trained incarcerated people and staff. Staff psychologists must create an individualized treatment plan for anyone held for more than 5 days that allows for daily out-of-cell time and participation in structured activities.

#### Transforming “solitary confinement” into the behavioral intervention unit

Following their return from Norway, ND DOCR reconfigured some NDSP cells built during the 2013 solitary confinement expansion into “preferred housing,” in which incarcerated people are housed in a single cell but are otherwise given access to programs and privileges available to general population prisoners. In 2016, the remaining cellblocks were converted into the “Behavioral Intervention Unit” (BIU) to be used as a “last resort” for people deemed to  need physical separation from the general population, usually for committing serious assaults.

The goal of the BIU is to reduce violence through mental health services guided by motivational interviewing, cognitive behavioral therapy, and positive psychology. Since its inception in 2016, the BIU committee—comprised of a Deputy Warden, behavioral health supervisor, chief of security, and unit manager—have conducted individualized, in-person reviews of each person’s case every 7 days including their behavior plan, engagement in programming, progress towards their goals, and the likelihood of violence if they return to general population. The review committee is required to provide a written explanation to each person stating a rationale for holding or releasing them from the BIU. In a transitional tier in the BIU, people are expected to continue to participate in counseling, but are allowed more property and privileges and eat meals and recreate with people from general population in preparation for returning to general population.

ND DOCR policy states that the BIU “offers residents as much meaningful out of cell time and enrichment activities as possible to minimize distress and isolation to people living in the unit.” Both staff and incarcerated people described ways in which the BIU is less harsh than the solitary confinement units were before reforms, including increased counseling, more meaningful out-of-cell time, use of privileges and property to reinforce positive behavior, and better interactions between staff and incarcerated people. One imprisoned person said, “I just got out of there … Now, it doesn’t even feel like punishment.” Releases from BIU are guided by staff’s efforts to recognize positive behaviors rather than punish undesired ones. One official explained:

It used to be that when it came time to consider release from restrictive housing, we had to rely mainly on the fact that a person had not done anything “bad” during their stay in restrictive housing. But they also did not have any opportunities. Now, we don’t rely only on the absence of negative behavior, and instead focus on the presence of positive behavior. With that, the mantra “progress not perfection” is something we remind ourselves of often.

Both staff members and individuals who had experienced BIU firsthand reported that people were no longer being kept in their cells for 22–23 hours or more each day, and now were allowed more personal property (e.g., televisions, reading materials), programming options, and family communication. Mental health professionals were given a larger role in unit operations as well as interacting with incarcerated people more frequently and conducting group therapy sessions three times per week.

To further limit BIU stays for persons who continuously violate minor rules in the general population, officers are now required to summarize the alternative sanctions short of confinement that have been applied, articulate the expected benefits of placing the person in the BIU, and articulate individualized plans that incorporate positive reinforcement strategies to address negative behaviors. In addition, the warden is now required to review each case of every person who has been held “continuously” in the BIU for the previous 4 months. Any BIU placement that reaches 12 months must be reviewed by the ND DOCR director.

Despite these policy changes, some ND DOCR officials have acknowledged that long BIU stays remain a pressing concern. According to administrative data, 445 unique individuals spent at least 1 day in the BIU between 2018 and 2019, of which 5.39% spent between 100 and 180 days, 4.04% between 180 and 365 days, and 0.9% more than 1 year. Incarcerated people shared mixed reactions to the BIU’s cognitive behavioral therapies and skills training. Some reported that they had experienced clear benefits. For example, one person said that engaging in skills training “has helped me in the long run. I’m more cool-headed. I’ve developed that time to slow down and actually think before reacting.” Others observed that the usefulness of skills training tended to diminish after leaving the BIU, because people are no longer supervised by the more knowledgeable and better trained BIU staff. They felt officers working in general population units sometimes fail to appreciate their attempts to use the skills that they had developed in the BIU, and some general population officers act more abrasively towards them than when they were posted in the BIU. As one incarcerated persont told us:

The whole program’s gone, once you get out. COs act one way back in BIU, and they come out and they start working in the West, and they’re a whole other CO. They’re no longer how they were taught, back there.

Although the BIU was described by incarcerated people and staff as being less isolating and punitive than before the reforms were implemented, the people who are confined there continue to experience significant day-to-day restrictions (e.g., they are restrained when leaving their cell, eat meals and recreate alone, and are allowed limited property). In addition, as one NDSP staff member noted, living conditions in the BIU remain bleak, “it’s just the staleness of the area and the cages, no greenery. Many of the things that we shouldn’t have … is what it [BIU] is.”

Officers and clinicians candidly acknowledged that, despite reforms, the level of social isolation that is still being imposed in the BIU was problematic and likely had adverse effects on some people. They reported that some incarcerated people do not benefit from the skills training and "behavior modification plans", and instead deteriorate mentally during their time in the unit. A BIU officer who provides skills trainings said, “With some individuals, when they come back here, they’ll stop taking their meds and that kind of sends them backwards.” An individual who had been placed in BIU multiple times found the positive-reinforcements and cognitive-based skills training "superficial" and ineffective and said the conditions in the BIU were still socially isolating and dehumanizing. He stated: “The less human you treat me, the less human I’m going to behave. I want to be treated like a human. I want to have those things that make me comfortable. Without those things, I can’t be who I want to be.”

Other officials recognized the BIU, and other reforms, were works-in-progress, subject to structural forces in the larger prison system that were beyond their control (such as overcrowding and financial constraints) that prevented them from moving as fast or far as they would have liked. One official stated, “We’re not out of the woods yet. Being in BIU can still have a negative effect. I don’t think we do enough out-of-cell time or provide enough interventions to counteract what is still fairly isolating.” A person imprisoned at NDSP who had spent time in the BIU echoed this sentiment, “I think we’re close here, but there’s going to be 15 very important parts and we probably have 10 of them whereas there used to be about two of them.”

#### Changing the nature of the correctional officer role

Following their return from Norway, ND DOCR officials also took steps to transform the nature of the correctional officer role by explicitly incorporating the Norwegian Correctional Service principles of “dynamic security,” “normalization,” and “progression.” (See Table 1.) The new correctional officer role included an explicit commitment to build trust and positive relationships with incarcerated people, to treat them with respect, and to focus on the reinforcing positive behaviors, rather than punishing undesirable ones. To socialize and instill officers in this new role, ND DOC officials instituted department-wide trainings in dynamic security, motivational interviewing, and mediation, and encouraged staff to apply these principles and tools to proactively resolve conflicts rather than resorting to solitary confinement. In the BIU, for example, psychologists began training officers to administer individually tailored positive reinforcements and BIU officers were instructed to practice newly acquired cognitive and behavioral skills with each person on the unit at least once per shift.

As part of the process of modifying the nature of the correctional officer role, staff were trained to use mediation in response to physical altercations to help those involved identify and reconcile the issues leading to conflict. Although altercations and fights between incarcerated persons still sometimes precipitate immediate physical separations (in the BIU or SAU), they now typically last for no more than 1–3 days, until mediation is completed. At that point, people are usually returned to the general population. Incarcerated persons described the benefits of the mediation process as much preferred to solitary confinement. According to one individual:

Say him and I got into a fight … they will make us sit and talk to each other with two other staff members. It’s pretty much just to clear things out and make sure... there is not going to be animosity. People cool down, shake hands and forget about it. And I’d say, nine out of ten times that’s exactly what happens, everything’s fine.

Staff reported that they came to regard mediation as an effective response to altercations that previously would have resulted in time spent in solitary confinement. A case manager explained:

We’ll give them [incarcerated people] the chance to speak both sides, to talk, and explain what they were thinking, what they were feeling at the time. I can’t even think of the last time that we had a mediation between two people who fought that still chose to fight again afterwards.

#### Reconfiguring correctional officer training 

The ND DOCR modeled its new recruitment and training protocols on those of the Norwegian Correctional Service. As one correctional official explained, “As we got more formal about [reform], I came to realize that a lot of the stuff we were doing would never be sustainable in the pouring rain if we did not embed it into our training.” Therefore, the department developed system-wide trainings to enhance officer skills in motivational interviewing, positive reinforcement, and dynamic security and simultaneously educated them on the psychologically harmful effects of solitary confinement. Staff reported that these trainings not only were important for increasing officer engagement but also gave officers a framework with which to understand why people sometimes deteriorated psychologically and engaged in self-harm while in solitary confinement. An officer explained:

So, we have a person that’s back here [BIU] right now that’s been back in segregation off and on through my entire career and before. And even people that I came up with, they’re even starting to see, wow … something’s going on with him. He wasn’t like this before. And I can actually point to look how many times he’s been in Segregation. Look at the duration that he was in Segregation. I can explain to them what happens with the brain if you’re in Segregation with limited stimulus. More and more people are starting to see that now.

The department also revised its job descriptions and recruitment strategies to be more consistent with the Norwegian approach, making it clear at the outset that they were seeking potential employees who were committed to rehabilitation. In order to teach the panoply of additional skills necessary to perform in the new correctional officer’s role, and to enhance professionalism in the officer corps, the length of the new officer training program was significantly increased—from 3 weeks to 6 months. In describing the kind of person the department now sought to hire in the new correctional officer role, one official said:

We don’t really want cops. Rather than an authoritarian, we want people who are more of the educator, social worker, behavioral health person – someone that just wants to work with people.” A case manager expressed the hope that these organizational changes would bring “new generations of officers that are a lot more receptive to reforms … I’m looking for someone who can communicate well, who can leave their ego at the door, and not hold grudges.

### The impact of policy changes on the ND DOCR’s use of solitary confinement

**Fig. 3:**
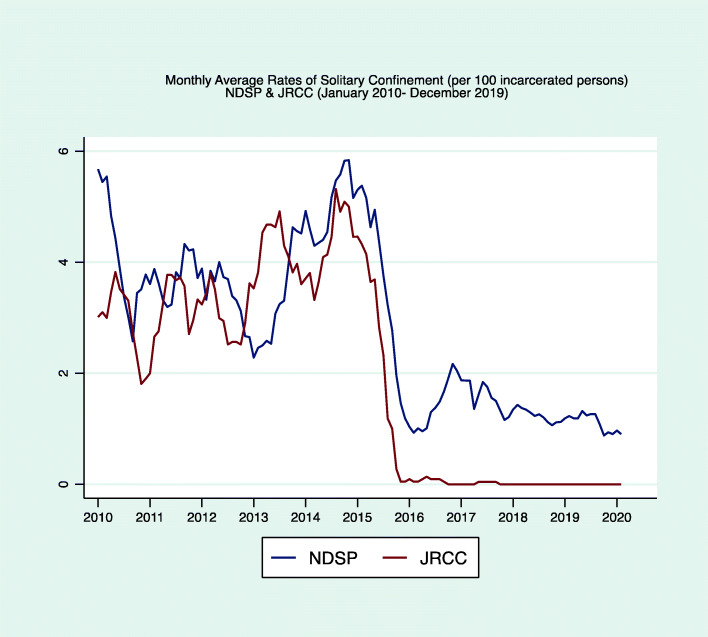
plots the monthly rates of solitary confinement placements (per 100 incarcerated persons) at NDSP (blue) and JRCC (red) from January 1, 2010 through December 31, 2019. It illustrates the sharp decline starting in the Fall of 2015 when North Dakota started implementing the policy changes described in this manuscript

ND DOC officials reported that the host of Norway-inspired policy changes that they implemented, as we described above, helped them achieve dramatic reductions in the numbers of persons housed in solitary confinement. Specifically, between January 2016 and December 2019, the ND DOCR reduced the total number of people held in solitary confinement-type units (placements in the SAU or BIU) in NDSP and JRCC by 74.28% (compared to the pre-reform period, January 2010 through December 2015). The monthly rate of solitary confinement sanctions decreased by 99% at JRCC and 59.1% at NDSP over this same timeframe (Fig. 3). Most admissions into SAU or BIU were shorter than before the reforms were implemented. In 2015, the median length of stay for people housed in a cell designated for administrative segregation was 89 days; it dropped 59%, to 34 days, over the next 4 years (between January 2016 and December 2019).

In addition to these reductions in the frequency and duration of solitary confinement, ND DOC officials and staff reported that Norway-inspired policy changes helped them modify other aspects of the way their prison system operated. We describe the most important of these modifications below.

### Reductions in the number of people with serious mental illness in solitary confinement

Many officers reported that the increased clinical services that were now being provided to incarcerated people—ones in accord with the Norwegian principle of “progression” that continuously seeks to move people who are incarcerated to better, less restrictive living conditions — has contributed to significant reductions in the number of people with serious mental health needs housed solitary confinement. A former Warden explained:

We have people right now that we never thought would stay out of a segregation unit. For years, 10 years, 12 years, back and forth between segregation, general population. Many of those guys today, since the changes have happened in restrictive housing, they’ve never come back.

**Fig. 4:**
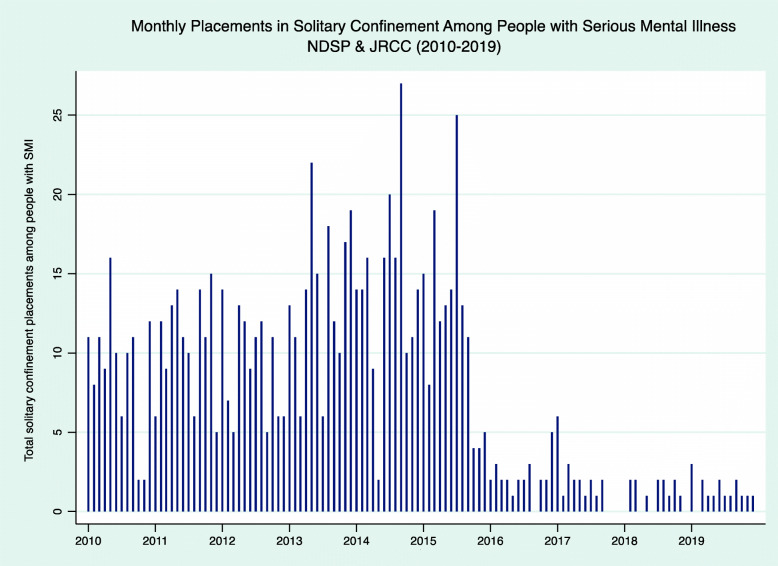
displays the total number of incidents each month (between January 2010 and December 31, 2019) in which an individual with a diagnosis of serious mental illness (i.e. schizophrenia, bipolar disorder, major depression, and/or PTSD) was placed in solitary confinement (i.e. sanctioned to disciplinary segregation or referred to administrative segregation) following a rule violation conviction. It shows a significant decrease beginning at the end of 2015 and a total reduction that was sustained through the post-reform observation period

From 2016 through 2019, the ND DOCR achieved a substantial decline in the number of solitary confinement sanctions imposed on people with a documented history of serious mental illness (i.e., schizophrenia, bipolar disorder, major depression, and/or PTSD), Fig. 4. For instance, before reforms were implemented (between January 1, 2010 and December 31, 2015), there was an average of 11.39 solitary confinement placements among people with serious mental illness per month between both prisons. In the post-reform period (January 1, 2016, to December 31, 2019), it decreased 630% to an average of 1.56 placements per month,. Clinical staff attributed this reduction to more in-depth mental health screenings, enhanced mental healthcare in general population, and the option to divert people with serious mental illness to the more treatment-oriented SAU. One clinician noted, “there is even more awareness that someone with a serious mental illness shouldn’t be in [solitary confinement].” Staff and incarcerated people said that mental healthcare has improved in SAU alongside declines in self-harm events and less violence against staff. The 24 treatment beds in the SAU mostly remain at full capacity, occupied by many individuals who, in past times, would have been placed in solitary confinement.

Most participants agreed that mental health services have improved, noting that psychologists now handle situations that previously were addressed by security staff. One imprisoned person noted, “the treatment department is empowered to work with people like humans.” Several acknowledged the role of the behavioral health staff in helping long-term residents progress out of isolation. Another person who spent 28 out of 30 years incarcerated in solitary confinement said:

I went all those years without the treatment department working with me. For a long time, they didn’t have my meds straightened out … I was feeling I had to act out or whatever. But now it’s easier to deal with things. They’re actually going out of their way to work with you now.

Staff also acknowledged the value of another Norway-inspired approach—giving incarcerated people  who had successfully transitioned to the general population after solitary confinement a specialized role as “peer mentors,” to help support other incarcerated peoples improve their problem-solving and coping strategies to avoid future BIU placements. One staff member noted that “having 45 people that take care of other residents in some fashion” has greatly enhanced access and quality of behavioral health services, while improving the well-being of the peer mentors themselves. Another official explained:

They’ve [peer mentors] become part of the team. That wouldn’t have ever happened without the foundation of dynamic security and coming in and saying it’s normal for people to take care of people. We’ve seen huge, huge changes in people; not only the people that are being cared for but the guys, the mentors. All of a sudden, they have purpose. They have hope. It just completely changes their outlook.

#### Improved staff-incarcerated persont interactions

Both staff and incarcerated people acknowledged that their interactions with one another had improved in the wake of these overall reforms. ND DOCR officials attributed some of the improved interpersonal interactions to increased staff awareness about the psychological harms of solitary confinement, especially for  those individuals who had long histories of solitary confinement and/or BIU enrollment. Staff also reported enhanced job satisfaction, reduced day-to-day stress, and increased perceptions of personal safety. An officer credited the infusion of dynamic securityprinciples into department policy, training, and daily practice as leading to a less volatile environment at NDSP: “It is calmer. Staff talk to people all the time. We’re stopping many, many incidents before they happen because there’s a rapport. I’ve seen so much change in some of the guys that have been here for a long time.”

Clinicians and administrators also observed the benefits to staff that accrued from working in a less stressful and noxious environment in the SAU, where many fewer cell extractions took place. One stated “it’s a more positive experience now. It doesn’t smell, it’s not loud, there’s not SORT [Special Operations Response Team] coming in every other day. It’s just a different vibe.”

In addition, staff reported that there was less hostility and conflict between them and people assigned to the BIU, compared to the administrative segregation units of the past. They attributed these changes to overall reductions in the use of solitary confinement and the fact that even those person who were housed in BIU now had more opportunties for human interaction andtime out of their cell. An NDSP staff member noted that the changed policies and practices had drastically decreased deployments of SORT and crisis teams in the BIU:

It’s an amazing thing. Our special operations teams in our segregation unit are basically zero. Our crisis negotiations teams, if we have a situation, all of those things have been impacted … you actually start forgetting about it, because it just doesn’t happen.

Similarly, a clinician described the benefits of the new dynamic security approach this way: “[There is now] a lot more relationship building, more talking to people when they’re struggling, intervening sooner, working through prevention, and being flexible around things that we used to be very static about.” These transformations in the interactions between staff and incarcerated people fostered what many of the persons whom we interviewed described as a deeper change—the creation of a more compassionate culture in the units themselves. One clinician stated:

For me personally, the measure of success that I see is the actual growth and change that we’ve seen in the residents. Those things are harder to measure. But the fact that we had guys who were just consistently angry, able to feel good, and let go of some of that anger, and just have moments where they are enjoying life despite being locked down is the big success.

Staff also reported that being trained to better help people solve personal problems, understanding the traumas and adversities endured in their life histories, and witnessing their improvements following the policy changes had a positive impact on their job and perspective of incarcerated people. A case manager, initially skeptical of the reforms, said:

It’s made my working relationships with the residents... and actually even with the staff... a lot better. I don’t take things to heart or look at it as an attack on me. I just look at it as what it is. It’s a behavior. Behaviors are learned. Behaviors can be unlearned. Let’s find out why this happened and try to figure out a better way.

Many staff reported discovering a deeper sense of responsibility and purpose in their profession. One BIU officer emphasized the importance of empathy, respect, ;and professionalism to counteract the “us versus them” mentality that pervades correctional culture. Another BIU officer explained:

A lot of times when they come down here, excuse my language, they’ll just say “you’re just a fucking blue shirt, I don’t trust you.” But you just show them respect or show them human decency. People that come down here just crave respect and a conversation. Like an intelligent conversation. Regardless of what shirt I’m wearing, regardless of what you think I represent, I’m here to help. So, the one-on-one rapport and being able to see that change, just little by little. That is rewarding.

Incarcerated people agreed that many officers became less punitive and more skilled at resolving conflicts through mediation. They confirmed that many infractions that previously would have resulted in solitary confinement, particularly minor fights or conflicts, now do not. One man, imprisoned for 17 years, said:

In the past, let’s say you got into a fight, you were in the hole for 30 days, maybe the first time and then the second time, six months or a year in a lockdown situation. Now, it’s a lot less time in the hole. They just make sure that the situation is not going to get out of control. It might only take a day or two, and you’re not just locked into a cell.

Staff also reported that incorporating dynamic security into their policies and practices has resulted in a positive change in workplace culture. One officer explained:

Now staff sit down, think, and talk thoroughly about the positive things happening. That shifts staff culture and way that people see their jobs a lot. It breaks through some of that negative mindset that can happen when constantly dealing with situations where you feel a lack of control and turn to fear-based responding.

A clinician illustrated one of the ways that incorporating dynamic security into ND DOCR’s practices had changed the staff perceptions of workplace safety: “You will hear staff say things though like, ‘It’s not the cuffs that keep you safe, it’s the relationship you have with a person,’ and that type of thing. And it’s just a really great principle that’s spread across the whole entire facility.”

#### Trends in institutional violence following solitary confinement reforms

As noted, ND DOCR’s Norway-inspired policy reforms were initially met with skepticism by some staff members and officials who feared that the changes would lead to increased violence and ultimately endanger staff. One clinician described colleagues as being, “very worried that doing anything that’s not punitive or authoritarian is going to end up with them hurt.” Similarly, one security official recalled:

There was this narrative that we were going to make things more dangerous in the general population by not allowing people to be locked up in solitary confinement. And that was something that we really had to show in the data, really keep track of violence and fights and acknowledge the staff’s fear.

However, contrary to these expectations, most of the staff and incarcerated people whom we interviewed agreed that the reforms appeared to them to have either reduced violence levels or that they remained the same. Both groups reported that assaults resulting in serious injury had been reduced. Some incarcerated people did not perceive any difference in the number of assaults against staff, stating that people with histories of assaulting staff are typically the ones who still engaged in that behavior. One individual said, “I don’t necessarily think there [are] less staff assaults. It’s not like random inmates [sic] are beating up [correctional officers] now, it’s the same people.”

#### Overall rule infractions for violence across both prisons

As noted, ND DOCR’s Norway-inspired policy reforms were initially met with some skepticism by some staff members who feared that the changes would lead to increased violence and ultimately endanger staff. One clinician described colleagues as being, “very worried that doing anything that’s not punitive or authoritarian is going to end up with them hurt.” Similarly, one security official recalled:

There was this narrative that we were going to make things more dangerous in the general population by not allowing people to be locked up in solitary confinement. And that was something that we really had to show in the data, really keep track of violence and fights and acknowledge the staff’s fear.

However, contrary to these expectations, most staff and incarcerated people reported that the reforms appeared to them to have either reduced violence levels or that they remained the same. Both groups reported that assaults resulting in serious injury had been reduced. Some incarcerated people did not perceive any difference in the number of assaults against staff, stating that people with histories of assaulting staff are typically the ones who still engaged in that behavior. One resident said, “I don’t necessarily think there [are] less staff assaults. It’s not like random inmates [sic] are beating up [correctional officers] now, it’s the same people.”

Our analysis of the ND DOCR’s data indicated that perceptions that violence decreased or remained the same following the reforms to solitary confinement were largely mirrored in the administrative data.

When we analyzed the department’s administrative records of disciplinary actions filed by correctional officers to quantify trends in interpersonal violence before and after reforms, we found that there were no statistically significant changes in the average monthly rates of all violent infractions (per 100 incarcerated persons ) across the two prisons before and after the reforms (fights between incarcerated people, assaults  between incarcerated people, assaults on staff, or overall, Table 2). The monthly rate of all combined disciplinary events involving violence across both prisons showed a slight and statistically insignificant decrease from 3.5 incidents per month before reforms (January 1, 2010, through December 31, 2015) to 3.44 per month following the reforms (January 1, 2016, through December 31,2019) (*p* = 0.66). There also were no statistically significant changes in the monthly rate of assaults on staff following the reforms (0.24 vs. 0.32 events per month, *p* = 0.203), nor in assaults between incarcerated people (0.70 vs. 1.16 assaults per 100 incarcerated persons , *p* = 0.31).

**Table 2:** Facility-wide Monthly Rates of Disciplinary Events Involving Physical Violence (Per 100 incarcerated persons ) NDSP, JRCC, & Combined Pre-reform (January 2010–December 2015) vs. Post-Reform (January 2016–December 2019)

	NDSP		JRCC		Combined	
Indicator	Monthly Mean	***p***-value	Monthly Mean	***p***-value	Monthly Mean	***p***-value
**All Violent Infractions**						
Pre-reforms	2.85		2.17		3.50	
Post-reforms	3.49	0.46	1.98	0.00*	3.44	0.66
**Violence Against Staff**						
Pre-reforms	0.24		0.16		0.23	
Post-reforms	0.32	0.49	0.10	0.24	0.26	0.23
**Fights among Residents**						
Pre-reforms	1.29		2.91		1.89	
Post-reforms	1.98	0.04*	2.44	0.32	2.15	0.93
**Assaults between Residents**						
Pre-reforms	0.70		0.75		0.71	
Post-reforms	1.16	0.31	0.62	0.39	0.96	0.51

To further assess the relationship between changes in the use of solitary confinement and behavioral infractions for interpersonal violence, we calculated Incidence Rate Ratios (IRR) between the monthly rates of solitary confinement and each indicator of violence using negative binomial regression with fixed effects. Overall, there was a small, statistically significant, positive association between monthly rates of solitary confinement and overall violent infractions across both prisons (IRR = 1.012, *p* < 0.05), meaning that in months where the use of solitary confinement decreased there was a small and significant associated decrease in the monthly rate of violent infractions (Table 3).

**Table 3:** Incident Risk Rations from Negative Binomial Regression with Fixed Effects for Monthly Rates of Solitary Confinement & Violent Infractions (2010–2019)

	Incident Rate Ratio	95% CI		*p*-value
**NDSP**				
All Violent Infractions	1.0001	0.992	1.009	0.886
Violence Against Staff	1.003	0.980	1.026	0.781
Fights between incarcerated people	0.995	0.983	1.006	0.363
Assaultsbetween incarcerated people	0.995	0.979	1.011	0.551
**JRCC**				
All Violent Infractions	1.034	1.019	1.052	0.00*
Violence Against Staff	1.050	1.016	1.085	0.003*
Fights between incarcerated people	1.026	1.006	1.047	0.011*
Assaults	1.011	0.985	1.037	0.412
**Combined**				
All Violent Infractions	1.012	1.005	1.019	0.001*
Violence Against Staff	1.016	0.999	1.033	0.064
Fights between incarcerated people	1.006	0.997	1.016	0.150
Assaults between incarcerated people	1.003	0.990	1.015	0.683

#### Rule infractions for violence at NDSP

**Fig. 5:**
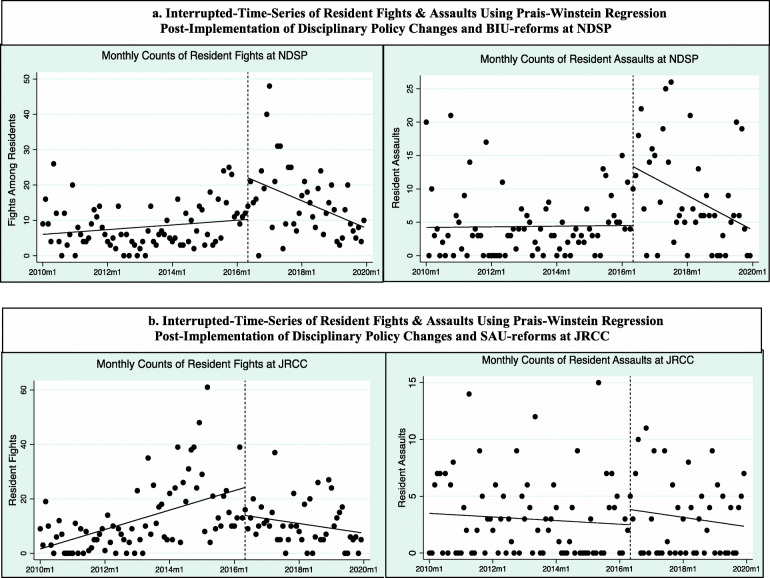
**a** shows actual and predicted linear trends in the monthly counts of fights (left) and assaults (right) at NDSP, before and after ND DOCR implemented changes to limit use of solitary confinement in response to rule violations and the established the Behavioral Intervention Unit (BIU). The vertical line denotes the intervention point of May 1, 2016, when these reforms were operating and was selected in consultation with ND DOCR leadership. The graph on the lefts suggests that monthly assaults increased from January 2010 to May of 2016 but did not reach statistical significance (*Β* = 0.06, p = 0.221, CI = − 0.03, 0.14). It also shows that during the early months after reforms, fights increased significantly (*Β =* 11.78, *p* > 0.0001, CI = 5.46, 18.11). However, the downward slope of line after the intervention point suggests a significant overall decrease in the monthly trend of fights at NDSP between May 2016 and the end of 2019 (*Β =* − 0.38, *p* > 0.0001, CI = − 0.60, − 0.16). The nearly flat slope of the pre-intervention line on the graph to the right that suggests monthly assaults did not change significantly between January 2010 and May of 2016, (*Β =* 0.004, p = 0.893, CI = − 0.54, 0.06). As with fights, this plot also shows that during the early months after reforms, assaults between incarcerated people increased significantly (*Β =* 8.80, *p* > 0.0001, CI = 4.57, 13.04). However, the downward slope of line after the intervention point suggests a significant overall decrease in the monthly trend of assaults at NDSP between May 2016 and the end of 2019 (*Β =* − 0.22, *p* > 0.001, CI = − 0.37, − 0.07). **b** shows actual and predicted linear trends in the monthly counts of fights (left) and assaults (right) at JRCC, before and after ND DOCR implemented changes to limit use of solitary confinement in response to rule violations and enhanced staffing and services in the Special Assistance Unit (SAU), a housing area for people with acute psychiatric needs. The vertical line denotes the intervention point of May 1, 2016, when these reforms were operating at JRCC and was selected in consultation with ND DOCR leadership. For the graph on the left, the upward slope of the prediction line, before the intervention, suggests that monthly fights increased significantly prior to May of 2016 (*Β =* 0.30, *p* < 0.0001, CI = 0.19,0.39). By contrast, the downward slope of line after the intervention point illustrates a significant decrease in the monthly trend of fights in the initial months, post-reforms (*Β =* − 10.32, *p* > 0.0001, CI = -0.69,-0.19), and a significant monthly decrease from May 2016 through December 2019 (*Β = −* 0.45, *p* > 0.01, CI = − 0.69, − 0.19). For the graph on the right, the downward slope of the line, before the intervention, suggests that monthly assaults decreased prior to reforms, but was not statistically significant ((*Β =* − 0.13, *p* = 0.377, CI = − 0.43, 0.02). Similarly, the downward slope of the line after the intervention point illustrates a small increase in assaults in the initial months post-intervention (*Β =* 1.34, CI = − 0.80, 3.48), followed by a decrease in the monthly trend of assaults in the post-reform period (*Β = -0.21 p* = 0.574, CI = − 0.9, 0.05) that was not statistically significant

At NDSP, we found that fights between incarcerated people  (the lower severity level of rule infractions involving violence) increased across all housing units from over 1 to just under 2 incidents per 100 incarcerated persons per month 1.29 vs. 1.98, *p* < 0.001), Table 2. This finding appears to have been driven by an increase in fights which occurred in the first year after the reforms were implemented in 2016, followed by a downward trend for the remainder of the observation period, Fig. 5a. It is important to note that the BIU was not operational and revised disciplinary policies were not in effect until May of 2016, when the downward trend began.

Figure 5a shows actual and predicted linear trends in the monthly counts of fights (left) and assaults (right) at NDSP, before and after ND DOCR implemented policy changes to limit use of solitary confinement in response to most rule violations and the established the Behavioral Intervention Unit (BIU). The vertical line denotes the intervention point of May 1, 2016, when these reforms were operating and was selected in consultation with ND DOCR leadership.

For the graph on the left, the upward slope of the prediction line, before the intervention, suggests that monthly assaults increased from January 2010 to May of 2016 (*B* = 0.06, *p* = 0.221, CI = − 0.03, 0.14), but not to a statistically significant degree. It also depicts that during the early months after reforms, fights increased significantly (*B* = 11.78, *p* > 0.0001, CI = 5.46, 18.11). However, as the downward slope of line illustrates, following these policy changes there was a significant overall decrease in monthly trends of fights at NDSP, between May 2016 and the end of 2019 (*B* = − 0.38, *p* > 0.0001, CI = − 0.60, − 0.16). For the graph on the right, as the nearly flat slope of the pre-intervention line depicts, monthly assaults did not change to a statistically significant degree, between January 2010 and May of 2016, (*B* = 0.004, *p* = 0.893, CI = − 0.54, 0.06). As with fights, it shows that during the early months after these reforms, assaults between incarcerated people increased significantly (*B* = 8.80, *p* > 0.0001, CI = 4.57, 13.04). Similarly, however, the downward slope of line after the intervention point suggests a significant overall decrease in the monthly trend of assaults at NDSP between May 2016 and the end of 2019 (*B* = − 0.22, *p* > 0.001, CI = − 0.37, − 0.07).

Furthermore, when we examined the associations between monthly rates of solitary confinement and rule infractions related to violence following reforms, we found that none of the associations reached statistical significance (monthly rates of solitary confinement and resident fights, IRR = 0.995, *p* = 0.363); monthly solitary confinement and assaults between incarcerated people (IRR = 0.995, *p* = 0.551); and monthly solitary confinement and assaults on staff (IRR = 1.003, *p* = 0.781). The relationship between monthly solitary confinement and all violent infractions was also not statistically significant (IRR = 1.0001, *p* = 0.886) at NDSP).

The lack of an association between monthly rates of solitary confinement and infractions related to violence mirrored the perceptions of staff and incarcerated people expressed during interviews and focus groups in which neither group attributed the slightly increased average frequency of fights at NDSP to the solitary confinement reforms. Instead, most suggested that the increases were likely due to persistent overcrowding in the ND DOCR including, at NDSP, increased double celling, more crowded dining and recreational areas, and a lack of privacy and personal space over those years. For example, one incarcerated person  observed, “there’s some guys that just can’t have a cellie. They just can’t mentally or physically have that extra body inside with them.” Another noted that things at NDSP “got really violent because they doubled us up in the cell.” Others explained that over-crowding, combined with lack of programming, educational, and job opportunities, and reduced privileges resulted in constant idleness among general population. One person imprisoned at NDSP said that crowding has resulted in people without constructive options to spend most of their day locked-down and reduced yard time schedules. That is: “In locked down 18-plus hours a day. For rec time, more often than not, they’re [staff] late to let you out [of one’s cell], but they’re always on time to lock you back in.”

Incarcerated persons said that fights and assaults occur most frequently in the communal spaces of the East and West units, because people spend most of their day locked down and staff assigned to the unit is less experienced to meet their needs and constructively respond to grievances and conflicts. As another person imprisoned at NDSP explained,

I live in the East. I've lived everywhere in the institution. My unit is much more violent. They call it the ghetto. They treat it like that. The guards don't want to be down there. They always put the brand-new guards who don't know nothing. I don't care who you are but you're going to be slow at your job and all that breeds is that constant animosity, the screaming, everything else.

Another incarcerated person  lamented that crowding people who have spent the majority of their day idle in a cell with another person in a prison recreation space inevitably breeds tension and conflict.

I'm sorry, I don't care if we all get along or not. You put 200 dudes in a room that's meant for 50 people, there’s two benches and there's 25 people that want a [weight] bench. These people aren’t going to care that you don't bother nobody...You've been here for years doing the same routine. This guy’s come over and wants to work out too, who are you to tell him what to do. He's got nothing to lose.

Staff members also pointed to the overall overcrowding in the ND DOCR rather than solitary confinement reforms as being primarily responsible for the increased number fights in general population. One clinician said, “it takes a lot to live in a place that’s the size of a bathroom, with a toilet in it, and another person.” Similarly, an NDSP official attributed most of the additional “disruptive behavior” and even slight increases in violence to factors other than the solitary confinement reforms, including things over which the prison system had little control, such as sentencing laws, crowding-related declines in living conditions, and the expansion of NDSP in 2013. As this official put it:

It’s not just a prison problem. It’s a society problem. Over-incarceration … the lengths of sentences are too long. We expanded from 550 beds to 850 beds. We didn’t add any dining room space. We didn’t add any recreation space. We didn’t add any classrooms, no extra programming, and we actually cut the yard down by 40%. We just keep smashing people in. It gives people no privacy.

#### Rule infractions for violence at JRCC

At JRCC, fights were rising precipitously before reforms were implemented, but *dropped* substantially at the start of 2015 and reached a low point at the end of 2019, Fig. 5. These perceptions were mirrored in the JRCC administrative data analysis. While the monthly rate of solitary confinement sanctions decreased by 99% over the course of this case study, there were no increases in the average monthly rate of fights between incarcerated people,  between incarcerated people assaults, assaults on staff, at the facility level. Instead, rule infractions involving violence decreased significantly from just over 2 to just under 2 events per month per 100 residents (2.17 to 1.98, *p* < 0.05); the monthly rate of fights between incarcerated people, assaults between incarcerated people, and assaults on staff showed no statistically significant change (Table 2). We also found that with each additional decrease in the monthly rate of solitary confinement at JRCC, the rate of monthly violent infractions decreased by about 3.4% (IRR = 1.034, *p* < 0.05), with about a 5% decrease in staff assaults (IRR = 1.050, *p* < 0.05) and a 2.6% decrease in fights between incarcerated people (IRR = 1.026, *p* < 0.05).

All of the incarcerated people  we interviewed at JRCC reported that the solitary confinement reforms had contributed to lower levels of hostility and violence throughout the prison. One focus group participant explained that when he first arrived at the prison “you couldn’t go a week without there being a fight. Now, they’re few and far between. It’s chilled out quite a bit. People get along more, a lot more social interaction.” Another noted that disciplinary policy changes and improvements in how officers interact with incarcerated people  “has made less people angry, more people happier, there’s a better vibe here and as a result there’s just been less violence and no stabbings here.”

Figure 5b shows actual and predicted linear trends in the monthly counts of fights (left) and assaults (right) at JRCC, before and after ND DOCR implemented changes to limit use of solitary confinement in response to rule violations and enhanced staffing and services in the Special Assistance Unit (SAU), a housing area for people with acute psychiatric needs. The vertical line denotes the intervention point of May 1, 2016, when these reforms were operating at JRCC and was selected in consultation with ND DOCR leadership. For the graph on the left, the upward slope of the prediction line, before the intervention, suggests that monthly fights increased significantly prior to May of 2016 (*B* = 0.30, *p* < 0.0001, CI = 0.19,0.39). By contrast, the downward slope of line after the intervention point illustrates a significant decrease in the monthly trend of fights in the initial months, post-reforms (*B* = − 10.32, *p* > 0.0001, CI = -0.69,-0.19), and a significant monthly decrease from May 2016 through December 2019 (*B* = − 0.45, *p* > 0.01, CI = − 0.69, − 0.19). For the graph on the right, the downward slope of the line, before the intervention, suggests that monthly assaults decreased prior to reforms, but was not statistically significant (*B* = − 0.13, *p* = 0.377, CI = − 0.43, 0.02). Similarly, the downward slope of the line after the intervention point illustrates a small increase in assaults in the initial months post-intervention (*B* = 1.34, CI = − 0.80, 3.48), followed by a decrease in the monthly trend of assaults in the post-reform period (*B* = -0.21 *p* = 0.574, CI = − 0.9, 0.05) that was not statistically significant.

#### Rule infractions for violence in the BIU and SAU

Our analysis of the ND DOCR’s institutional data for the solitary confinement units themselves (the BIU and SAU) showed reduced levels of violence following the reforms. For example, we found that physical violence occurred less frequently in the BIU than it had in the NDSP solitary confinement units before reforms were initiated. Specifically, in the 4 years before the Norway-inspired reforms were implemented (January 1, 2012, through December 31, 2015), there were 53 assaults on staff in the solitary confinement units at NDSP, charged against 13 different people. By contrast, no staff assaults were reported during the first 2 years of the BIU program. Although staff assaults increased to 21 in the BIU over the next 2 years, most (85.7%) involved just one incarcerated person. Assaults between incarcerated people also decreased after the BIU reforms were implemented: a total of 17 such assaults occurred between 8 individuals from 2012 through 2015 in the traditional solitary confinement units, as compared to 10 such  assaults involving 3 individuals  in the BIU post-reforms.

When asked about violence in the BIU, a security official said “it ebbs and flows. There’s times where we’ll have very little going on, and then there’s other times where it seems like the roof’s caving in.” Another NDSP staff member perceived fewer BIU emergencies than occurred in the solitary confinement units of the past:

All of a sudden, the trauma and emergencies, the things that happen on a daily basis just continue to reduce specifically in that unit. You’re still going to have bad days, but the bad day today is different than the bad day it was [before].

There were also perceived and measurable decreases in disciplinary incidents involving physical violence in the SAU following reforms. Monthly average incidents involving violence in the SAU decreased by 52.17% and were significantly lower post reforms (1.15 to 0.55 per month, *p* = 0.00). The monthly rate of staff assaults decreased by 62.82% (0.78 to 0.29, *p* < 0.05).

#### Behavioral infractions for violence among peoples with serious mental illness

We also found reductions in rule infractions for violence by persons with serious mental illness (SMI) diagnoses across both NDSP and JRCC. In the pre-reform period—from 2010 to 2015— 28.02% of all violent rule infractions involved a person with an SMI diagnosis. This decreased to 14.95% in the post-reform period, despite an increase in the total number of people diagnosed with SMI in the prison population.

Clinicians and security staff reported that multiple rincarcerated people with serious mental health needs who had previously spent most of their imprisonment in the SAU or in solitary confinement due to assaultive behavior were now living in dormitories without incident. One official said: “Guys who had never lived in GP, have been in GP for years, and are just doing so much better. And aren’t doing the assaultive behavior.” A mental health clinician for patients with severe mental health needs and histories of assaults reported that the changes within the SAU and the creation of BIU both helped people receive more intensive clinical services and stay out of segregation. She said that there were:

A bunch of people who we saw no movement on for a very long time, moved out and we got them out quickly and they have stayed out and done well. I just got a calendar notification about one of our guys who struggled for a long time going in and out [of solitary confinement] for violence and he’s been nonviolent for two years and is doing great.

## Discussion

This case study describes policy and programmatic changes made by the North Dakota Department of Corrections and Rehabilitation (ND DOCR) that included efforts to substantially reduce and ideally eliminate its use of solitary confinemen in the state's two largest prison. ND DOCR officials reported that these changes were inspired and guided by  public health and human rights principles  foundational to Norway's approach to public safety, ;during  their participation in the Amend program at the UCSF School of Medicine. Since their first visit to Norway in 2015, ND DOCR adopted diverse and interactive set of policychanges, an initiative ND DOCR called  *Increasing Humanity for People in Prison*, that resulted in  a 74.28% reduction in the overall use of solitary confinement

Prison staff and incarcerated people described how that thesystem’s previous heavy reliance on solitary confinement as a punishment for a wide array of rule violations  had profoundly adverse impacts on the health and wellness of incarcerated persons, a finding that echoes those in previous studies (Haney et al., 2020; Reiter et al., 2020; Smith, 2006;). Participants also acknowledged that working in these units contributed in a variety of ways to workplace burnout and stress among officers, including distress at witnessing the psychological deterioration of incarcerated people and feeling powerlessness to attend humanely and effectively to their serious needs. Their observations are consistent with, and contribute to, a growing literature on the role of carceral environments contributing to high rates of stress-related disease and early mortality reported among correctional officers (Finney et al., 2013; Morse et al., 2011; Regehr et al., 2019; Spinaris 2012).

Incarcerated persons and correctional staff alike described ND DOCR’s Norwegian-inspired policy changes as having improved the health and wellness of both groups. Among other things, the provision of enhanced clinical services for people with serious mental illness who engaged in disruptive and/or violent behavior reportedly helped to reduce the overall use of solitary confinement. So, too, did the implementation of policies designed to improve the frequency and quality of interactions between staff and incarcerated people. Staff reported enhanced job satisfaction, reduced stress, and increased safety in the wake of these reforms. Incarcerated persons and correctional staff both perceived the reforms as responsible for increased trust and less antagonism between the groups. Staff members also noted that the creation of a much-modified and enhanced correctional officer role, including the emphasis on the Norwegian practices of dynamic security (in which they proactively interacted with incarcerated people) and progression (in which staff helped incarcerated people move to less restrictive environments outside of solitary confinement), increased workplace satisfaction and provided officers with an elevated sense of purpose.

Contrary to the initial concerns of some staff members, who feared that the solitary confinement reforms, especially, would lead to major increases in violence, our analysis of institutional data indicated that this fear, by and large, did not materialize. We observed a statistically significant increase in the average monthly rates of fights among people imprisoned in the general population at one prison (NDSP) following the reforms (from just over 1 incident per 100 incarcerated persons   per month to just under 2 per month), however this level of violence in the lowest level of behavioral infractions for violence (fights as opposed to assaults) is not commensurate with the dramatic 74.28% reduction in the ND DOCR’s overall use of solitary confinement. In interviews, staff and incarcerated people alike perceived this increase in fights in NDSP’s general population as having been caused by factors *other* than the solitary confinement reforms, including overcrowding and an increased use of double-celling. Although it may be possible that some people who were released from solitary confinement contributed to this uptick in fights, when we examined the associations between monthly rates of solitary confinement and behavioral infractions related to violence, we found no statistically significant association between decreased use of solitary confinement and any indicator of violence, including at NDSP, suggesting that change in use of solitary confinement over this time was not a primary driver of increased fights. Furthermore, results from interrupted-time-series analysis indicates that at NDSP, once the BIU was operational and policy limiting the types of rule violations eligible for placement  in restrictive housing was enacted, there were initial increases in fights and assaults, followed by an overall downward trend for both infractions. By contrast, at JRCC, this analysis shows that following improvements to the SAU and aforementioned changes to disciplinary policy, initially fights declined and assaults increased. Though, as with NDSP, both trended downward over the post-reform period. Together, this analysis bolsters the perceptions of staff and people imprisoned at these facilities  that solitary confinement reforms were not followed by substantial or meaningful increases in violence, and that the benefits of reform outweighed any initial consequences.

We also found that staff and incarcerated peoplei perceived the policy changes as resulting in less tension between staff and incarcerated people, and overall improvements in the conditions within these prison environments. This perspective was born out in the analysis of administrative data in the unit designated for clinical mental healthcare (the SAU), where there were significantly fewer staff assaults following efforts to enhance clinical services and reduce isolation for imprisoned people with more severe psychiatric conditions. Such findings are consistent with other studies that have found that solitary confinement is an  ineffective and counterproductive long-term strategy for addressing violence in prison settings (Lovell et al., 2007; Luigi et al., 2020; Mears & Bales, 2009; Pizarro et al., 2014) and can take a  grave toll on the well-being of incarcerated people (Grassian, 2006; Haney, 2018b; Haney et al., 2020; Reiter et al., 2020; Rhodes, 2004; Smith, 2006; Strong et al., 2020). Moreover, some incarcerated people reported experiencing improvements to their psychological health and well-being as a result of North Dakota's efforts to minimize exposures to isolation while enhancing access to clinical and social services. Subsequent research should continue to examine potential benefits to the health and wellness of people directly affected by policies that reduce or seek to eliminate exposures to solitary confinement.

Of course, solitary confinement reform does not and cannot occur in a vacuum, any more than its increased use over the last several decades did. In fact, ND DOCR officials candidly acknowledged that, despite an ongoing  commitment to  continue reducing, and perhaps eventually even ending solitary confinement altogether, achieving and sustaining this goal was and is subject to structural impediments outside the control of ND DOC officials. In the recent past, those forces included legislative and judicial decisions to impose lengthy prison sentences, a lack of prison diversion programs in the state’s larger criminal justice system, social, racial, and economic inequities, ;and a short supply of resources to provide meaningful and equitable access to educational, vocational, and mental health services in communities with high incarceration rates as well as within the prison system itself. The challenges North Dakota’s Department of Corrections and Rehabilitation face are not unique to them and underscore the way in which the movement to end  the use of solitary confinement is interconnected with broaderefforts that are designed to reverse  society's reliance on  incarceration in response to complex social issues, while  embracing the values of human rights, ;dignity, ;and public health in interventions to abate deleterious prison conditions   (Ahalt & Williams, 2016; Haney et al., 2020; Lobel & Smith, 2019; Sakoda & Simes, 2021).

Our findings also have several limitations. We employed a case-study design to provide an in-depth description of North Dakota’s multi-dimensional efforts to decrease its use of solitary confinement. As a result, although we report on differences in the observed rates of violent rule infractions before and after reforms, our methods do not establish causal relationships between the two. We attempted to provide further context by conducting an analysis to explore the nature of associations between rates of solitary confinement and rule infractions involving violence over the study period and found that the large and sustained decreases in solitary confinement were not associated with significant increases in these measures of violence. In fact, at JRCC in particular, decreases in solitary confinement were significantly associated with decreases in violence. Future research should focus on examining whether and how specific policy components of solitary confinement reforms affect relevant indicators of institutional violence (e.g., use-of-force) and interpersonal violence (e.g., self-injury) while accounting for the potential interplay of other factors. Such studies might consider adopting more robust quasi-experimental designs, such as interrupted-time-series (Briggs et al., 2003; Labrecque, 2015) to estimate the effects of distinct policies more precisely on incarcerated people and staff by accounting for individual and institutional level confounders and covariates. Second, our qualitative findings were derived from participants chosen on the basis of their direct involvement in designing and/or implementing reforms (staff) or because they were directly affected by these policy changes (incarcerated people). Others who were not directly affected by the policies or involved in their implementation may have different perspectives. In addition, the retrospective nature of this study could lead to recall bias, however it is reassuring that the administrative data analysis largely corroborated our qualitative findings. Also, although we know of no specific reason to question the quality of ND DOCR’s institutional data, we cannot ensure its reliability and validity, as is often the case with correctional data that has not been collected explicitly for the purpose of research. Our use of the case study design, which includes analysis administrative data, qualitative data and policy analysis, minimizes reliance on non-research administrative data. Relatedly, although much of the institutional disciplinary infraction data we analyzed was quantitative in nature, it was not necessarily “objective.” That is, it was the product of interactions between staff and people in their custody  that were subject to staff’s interpretations (i.e., whether a person's actions  constituted an infraction and, if so, what kind and severity) that themselves might be influenced by other forces in the environment. Future studies should use qualitative methods or proactively collect data on rule violations to analyze infraction reports over time to assess their internal validity and reliability.

Finally, we would note that despite ND DOCR’s notable progress in significantly reducing the number of people exposed to solitary confinement and improving the living conditions to which they are subjected, staff and incarcerated persons  identified many remaining opportunities for continued improvement to the health and well-being of persons living and working in North Dakota prisons. Most notably, the BIU, which now functions as a unit of last resort that is mostly reserved for people who engage in serious acts of violence, is still a bleak and desolate environment described by both incarcerated people and staff as isolating and highly restrictive. Staff members continue to express concerns that it is likely to harmful to people who endure this type of environment, especially those who experience it on a prolonged basis. Moreover, there is always an ongoing risk that even a reform-oriented prison system like the ND DOCR eventually will regress to pre-reform practices, especially when discredited practices, such as solitary confinement, ;are viewed by prison officials as a necessary tool of last resort (Rubin & Reiter 2018). A commitment to sustaining and expanding the reach of the  policy changes that we have described, through ongoing monitoring of prison conditions and the status of policy changes that have been instituted, as well as implementation of additional legal and policy mechanisms to address remaining issues can serve as safeguards against potential reversals  (e.g., Haney & Pettigrew, 1986).

## Conclusion

This case study describes how participation in an immersive exposure to the Norwegian Correctional Service’s principles, policies, and practices helped to catalyze and guide one state prison system’s efforts to drastically reduce the use of solitary confinement and alleviate many of its worst effects by significantly modifying the way such units were structured and operated. Those efforts resulted in a host of positive changes in a range of policies and practices that were reported as beneficial to the  health and well-being of both incarcerated people and staff. The reductions in the use of solitary confinement were dramatic and, with the exception of one measure of low-level fights between incarcerated people  in the general population units of one of the prisons, the impact on infractions related to violence generally showed no change or actual improvements.

Decades of litigation, hunger strikes led by incarcerated people, and community-based advocacy have prompted prison systems to begin reducing their overreliance on solitary confinement (Ahalt & Williams, 2016; Earle, 2020; Gottschalk, 2015). In 2019 alone, 28 state legislatures introduced, and 12 passed, bills seeking to halt or limit the use of solitary confinement in prisons (Fettig, 2019). As these efforts continue and likely intensify, corrections officials, community advocates, and other stakeholders seeking to bring about significant solitary confinement reforms might benefit from the ND DOCR’s recent experience, which represents a set of initial steps that can be taken to alleviate at least some of the adverse consequences brought about by this practice.

## Data Availability

Data supporting the findings of this study are held by ND DOCR and is not publicly available due to privacy restrictions. Data may be available upon reasonable request to the authors and with permission of ND DOCR.
