# Correction to: “We just needed to open the door”: a case study of the quest to end solitary confinement in North ﻿Dakota

**DOI:** 10.1186/s40352-021-00159-1

**Published:** 2021-11-09

**Authors:** David H. Cloud, Dallas Augustine, Cyrus Ahalt, Craig Haney, Lisa Peterson, Colby Braun, Brie Williams

**Affiliations:** 1 Amend, University of California, School of Medicine, 490 Illinois Street, Floor 8, UCSF Box 1265, San Francisco, CA 94143 USA; 2 Department of Psychology, University of California, 1156 High Street, Santa Cruz, CA 95064 USA; 3 North Dakota Department of Corrections and Rehabilitation, 3100 Railroad Avenue, P.O. Box 1898, Bismarck, ND 58502–1898 USA

**Correction to: Health Justice 9, 28 (2021).**

**https://doi.org/10.1186/s40352-021-00155-5**

The article (Cloud et al., 2021), Cloud, D. H., Augustine, D., Ahalt, C., et al. (2021). “We just needed to open the door”: a case study of the quest to end solitary confinement in North Dakota. Health & Justice, 9(1), 1–25, was published on October 18th, 2021, before authors had received or approved a finalized proof. As a result, the original version had multiple typographical and formatting errors throughout the paper that have since been corrected.

The original article (Cloud et al., 2021) has been updated.
